# The Broad Clinical Spectrum of Metatropic Dysplasia: A Case Series and Literature Review

**DOI:** 10.3390/ijms26199783

**Published:** 2025-10-08

**Authors:** Kiabeth Robles-Espinoza, Eduardo Esparza-García, Juan Ramón González García, María Teresa Magaña-Torres

**Affiliations:** 1 División de Genética, Centro de Investigación Biomédica de Occidente, Instituto Mexicano del Seguro Social, Sierra Mojada 800, Independencia Oriente, Guadalajara 44340, Jalisco, Mexico; kiabethre@gmail.com (K.R.-E.); jrgg_gene@hotmail.com (J.R.G.G.); 2 Centro Universitario de Ciencias de la Salud, Doctorado en Genética Humana, Universidad de Guadalajara, Sierra Mojada 950, Independencia Oriente, Guadalajara 44340, Jalisco, Mexico; 3 Unidad Médica de Alta Especialidad, Hospital de Pediatría del Centro Médico Nacional de Occidente, Instituto Mexicano del Seguro Social, Belisario Domínguez 735, La Perla, Guadalajara 44360, Jalisco, Mexico; eduardoesparzagenetica@gmail.com

**Keywords:** metatropic dysplasia, spectrum of TRPV4-related dysplasias, Mexican patients, *TRPV4* gene, p.Asn796del, p.Pro799Leu

## Abstract

Metatropic dysplasia is an autosomal dominant skeletal disorder characterized by progressive kyphoscoliosis, severe platyspondyly, pronounced metaphyseal enlargement, and shortening of the long bones. This condition is caused by pathogenic variants in the *TRPV4* (Transient Receptor Potential Vanilloid 4) gene, which encodes a non-selective calcium channel involved in bone homeostasis. Variants in *TRPV4* have been associated with two major disease groups: skeletal dysplasias and neuropathies, with recent findings indicating an overlap in their clinical features. We report three patients with metatropic dysplasia, each presenting a distinct severity profile. All exhibited a bell-shaped thorax, significant platyspondyly, and shortened long bones with broad metaphyses. Notably, patients 1 and 3 had more complex clinical courses, including seizures and global developmental delay. Genetic analysis revealed two different *TRPV4* variants: p.Asn796del (patient 1) and p.Pro799Leu (patients 2 and 3). These cases illustrate variability in extra-skeletal manifestations, complications, and prognosis. In our patients with *TRPV4*-related disorders, the co-occurrence of neurological symptoms and skeletal abnormalities suggests a clinically heterogeneous spectrum consistent with a single disease rather than distinct entities. A comprehensive, multidisciplinary approach is essential to optimize management and improve the quality of life for patients.

## 1. Introduction

Metatropic Dysplasia (MD) (OMIM 156530) is a rare autosomal dominant skeletal disorder characterized by progressive kyphoscoliosis, severe platyspondyly, pronounced metaphyseal enlargement, and shortening of long bones. These features lead to a reversal of body proportions from birth to childhood, primarily due to trunk shortening [1]. Globally, MD is extremely rare; to date, fewer than 100 cases have been reported in the literature, and its prevalence is estimated to be under 1 per 1,000,000 [1].

Pathogenic variants in the *TRPV4* (Transient Receptor Potential Vanilloid 4) gene underlie MD and a spectrum of related disorders. In the skeletal system, TRPV4 variants cause both severe metatropic form and several milder spondylometaphyseal dysplasias (such as familial digital arthropathy-brachydactyly, Kozlowski- and Maroteaux type spondylometaphyseal dysplasia, and parastremmatic dysplasia). These variants have also been associated with neuromuscular conditions, including Charcot-Marie-Tooth type 2C (CMT2C), scapuloperoneal spinal muscular atrophy, and congenital distal spinal muscular atrophy [1]. Notwithstanding that *TRPV4*-related skeletal dysplasias and neuropathies were traditionally considered distinct, phenotypic overlap is increasingly recognized, reflecting a broad clinical variability even among patients with similar variants [2]. Most TRPV4-related disorders arise from gain-of-function missense variants, with no clear differences in the position or nature of amino acid changes between skeletal and neuropathic presentations [3].

The *TRPV4* gene, located at chromosome 12q24.11, encodes the transient receptor potential vanilloid 4 (TRPV4) protein, consisting of 871 amino acids. This protein is a non-selective calcium-permeable cation channel and belongs to the transient receptor potential (TRP) superfamily of ion channels [4]. TRP channels share a common structure that includes intracellular ankyrin repeat domains (at least one), a calcium/microtubule binding site, and six transmembrane regions [5].

TRPV4 functions as a tetramer, with four subunits forming a central ion-permeation core [6]. *TRPV4* is expressed in various tissues, including bone and brain [4,5,7], and plays essential roles in calcium-mediated membrane depolarization, osmo- and mechanotransduction, systemic volume regulation, bone homeostasis, arterial dilation, and nociception [5]. Given its broad tissue distribution and range of functions, pathogenic variants in *TRPV4* result in a wide spectrum of phenotypes.

Here, we report the first three Mexican patients with clinically and molecularly confirmed metatropic dysplasia, each exhibiting a different degree of severity and harboring distinct *TRPV4* variants.

## 2. Cases Description

### 2.1. Patient 1

A female patient was evaluated by a medical geneticist at 22 days old following a clinical diagnosis of thanatophoric dysplasia. A prenatal ultrasound at 37 weeks of gestation had revealed a cloverleaf skull, narrow thorax, and intrauterine growth restriction. She was the sixth pregnancy of healthy, non-consanguineous parents aged 33 (father) and 32 (mother). However, two previous pregnancies had ended in spontaneous abortion at 8 weeks of gestation, and one was ectopic.

**Figure 1:**
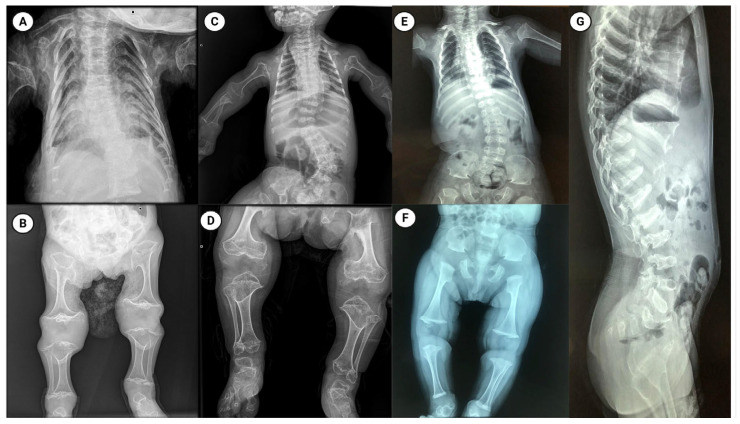
Radiographic features of metatropic dysplasia in three patients. Panels (**A**–**G**) show plain radiographs from patient 1 (**A**,**B**), patient 2 (**C**,**D**) and patient 3 (**E**–**G**). Thoracic and spinal images (**A**,**C**,**E**,**G**) reveal a characteristically bell-shaped thorax, marked platyspondyly (flattened vertebral bodies), and variable scoliosis. Appendicular images (**B**,**D**,**F**) demonstrate shortened long bones with pronounced metaphyseal widening/flaring and irregular metaphyseal contours. The radiographic phenotype is typical of metatropic dysplasia and illustrates inter-individual variability in severity across the three cases. Figure created with BioRender.

The patient was delivered via cesarean section at 37 weeks due to concerning prenatal findings. At birth, both weight (2800 g) and length (47 cm) were within normal ranges. Physical examination revealed dolichocephaly, a large anterior fontanelle, ptosis, muscular neck contracture, a bell-shaped thorax, bilateral camptodactyly in the hands, and mesomelia in the lower limbs. Radiographs showed a bell-shaped thorax, widened ribs, severe platyspondyly, and shortened long bones with broad metaphyses (Figure 1). Based on these findings, a diagnosis of MD was suggested, and physiotherapy was initiated to improve neck mobility.

**Figure 2:**
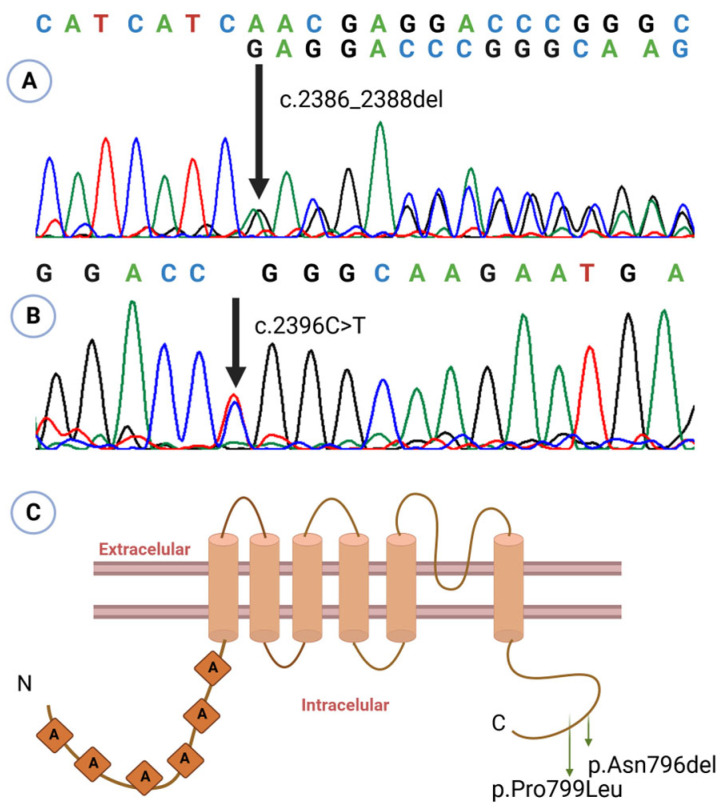
Sanger sequencing electropherograms showing the variants identified in patient 1 ((**A**): c.2386_2388del, p.Asn796del) and in patients 2 and 3 ((**B**): c.2396 C>T, p.Pro799Leu). The nucleotide change for each variant is indicated above the corresponding trace; overlapping peaks at the variant site indicate heterozygosity. (**C**) Structural localization of the identified variants on a schematic representation of TRPV4 derived from the model by Kang et al. (2012) [5]. Figure created using BioRender.

During follow-up, the patient developed multiple complications: (1) nasolacrimal duct obstruction, evaluated by ophthalmology; (2) partial seizures, treated with oxcarbazepine; (3) severe gastroesophageal reflux (GERD), requiring a gastrostomy at 8 months, with a cardiac arrest event during surgery; (4) neurogenic bladder, under ongoing evaluation; and (5) recurrent fever episodes following COVID infection. At examination, she presented with significant short stature (−5 SD) and low weight (−4 SD) for age. Brain magnetic resonance imaging revealed craniocervical junction narrowing with mild compression, and echocardiography showed a patent foramen ovale. Her overall prognosis was considered poor. Genetic analysis identified the variant p.Asn796del (c.2386_2388del; rs1889549195) in the *TRPV4* gene (Figure 2), classified as likely pathogenic. Parental testing was negative, indicating a de novo variant.

### 2.2. Patient 2

A female patient was referred for genetic evaluation following a prenatal diagnosis of a shortened and curved femur. She was born at 38 weeks of gestation, and both weight (2500 g) and length (48 cm) were normal. She was the first child of apparently healthy parents with no family history of skeletal disorders. His father was 28 years old and his mother 23. At physical examination, she exhibited restricted neck mobility, a narrow chest, pectus carinatum, and lumbar hyperlordosis. X-ray imaging showed a bell-shaped thorax, severe platyspondyly, and shortened long bones with broad metaphyses (Figure 1).

These clinical and radiographic characteristics were consistent with a diagnosis of MD. At follow-up, the patient showed improved neck mobility and no further complications. Her overall prognosis was considered favorable.

The pathogenic variant in *TRPV4* p.Pro799Leu (c.2396C>T; rs121912637) was identified in the patient (Figure 2). Absence of the variant in both parents confirms its de novo origin.

### 2.3. Patient 3

A one-year-old male presented with a history of intrauterine growth restriction and oligohydramnios identified by prenatal ultrasound in the 8th month of gestation. He is the only child of healthy, non-consanguineous parents aged 42 (mother) and 49 (father). He was delivered at 39 weeks with adequate weight and height for gestational age. At birth, a coccygeal skin tag was noted and surgically removed at two weeks of age. He showed global developmental delay and presented seizures at four months, currently managed with levetiracetam. On physical examination, he had normal height and weight, a wide forehead, upslanting palpebral fissures, microretrognathia, low-set ears, a short neck with limited mobility, a bell-shaped thorax, rhizomelic and mesomelic limb shortening, and restricted hip movement.

Computed tomography revealed spinal dysraphism at L5 and S1. Radiographic evaluation showed platyspondyly involving the cervical, thoracic, and lumbar vertebrae, metaphyseal widening, and shortening of the long bones, as well as irregular morphology of the carpal bones, astragalus, and calcaneus (Figure 1).

The patient experienced recurrent fractures, beginning with a left femoral facture at 2 years and 11 months of age following a fall from standing height. At 4 years, he sustained a fissure of the right femur of unknown origin, followed by diaphyseal re-fracture of the left femur at age 5. Bone densitometry at that time revealed a bone mineral density of −3.6 SD, prompting initiation of zoledronic acid therapy. At 6 years, he suffered a new fracture of the right femur; repeat densitometry showed improvement but persistent low bone mineral density (−2.5 SD). A second cycle of zoledronic acid was administered at age 7, resulting in normalization of bone mineral density. To date, no further fractures have occurred. Genetic analysis detected the same pathogenic variant as in patient 2: *TRPV4* p.Pro799Leu (Figure 2).

### 2.4. Genetic Testing Methods

Sanger sequencing of *TRPV4* exon 14 was performed for all patients. After obtaining informed consent, 2–4 mL of peripheral blood was collected from each participant. Genomic DNA was extracted using the DTAB-CTAB (dodecyltrimethylammonium bromide–cetyltrimethylammonium bromide) method [8]. Primers were designed with Oligo 6.0 software (Forward: 5′-GATTGCAGGCATGAGCCAC-3′; Reverse: 5′-CCAGGCATTCACAAGCAGC-3′), producing a 415 bp fragment. A touchdown PCR program was used with stepwise annealing temperatures of 62 °C, 60 °C, and 58 °C. All PCR reactions were carried out in a final volume of 10 µL. Amplicons were treated with 0.5 µL of ExoSAP-IT (Applied Biosystems, Waltham, MA, USA) for cleanup prior to sequencing. Sequencing reactions were performed in 10 µL volumes containing 100–200 ng purified PCR product, 0.5 µL of Ready Reaction Big Dye Terminator Kit v.3.1 (Applied Biosystems, Waltham, MA, USA) (Cat. 4337455), 1.5 µL of 5X sequencing buffer, and 2.5 pmol of primer. Thermal cycling consisted of an initial denaturation at 96 °C for 4 min followed by 25 cycles at 96 °C for 10 s, 55 °C for 5 s, and 60 °C for 2 min. Sequencing reaction products were purified on Sephadex G-50 columns (50 mg in 800 µL of water; sephadex medium or fine; Cytiva Life Sciences, Uppsala, Sweden). Capillary electrophoresis was performed on a SeqStudio genetic analyzer, and electropherograms were reviewed both manually and with SnackVar software (v. 2.4.3) [9], using reference sequence NM_021625.5. Variants were named according to the nomenclature recommended by the Human Genome Variation Society [10].

## 3. Discussion

Metatropic dysplasia is a rare and clinically heterogeneous skeletal disorder within the spectrum of TRPV4-related dysplasias. We report three unrelated patients, each with a de novo heterozygous *TRPV4* variant, who fulfilled the clinical and radiographic criteria for MD yet displayed markedly different phenotypes and degrees of systemic involvement. Together, these cases underscore that TRPV4-associated disorders are best conceptualized as a continuum of severity rather than as discrete, mutually exclusive syndromes.

TRPV4-related skeletal dysplasias frequently manifest prenatally or at birth and may be mistaken for other lethal or severe skeletal disorders, such as thanatophoric dysplasia [11,12], as occurred initially with patient 1. Accurate diagnosis, therefore, requires integration of detailed clinical and radiologic assessment with molecular testing. Patient 1 presented a complex MD phenotype characterized by significant skeletal abnormalities, severe gastroesophageal reflux, and neurological complications that impaired development and function. The variant identified in this case, p.Asn796del, is a likely pathogenic in-frame deletion that has been previously reported in at least one patient with Charcot-Marie-Tooth disease type 2C [13].

Patients 2 and 3 both carry the missense variant p.Pro799Leu, which has been associated with both mild and complex MD phenotypes, as well as with spondyloepimetaphyseal dysplasia Maroteaux type and CMT2C [1,13]. These two patients exemplify the clinical variability of the same variant: patient 2 exhibited a relatively mild skeletal phenotype with a favorable outcome and no major complications, whereas patient 3 had a more complex presentation that combined skeletal and neurological features, including developmental delay, seizures, and spinal dysraphism. In the literature, we identified seven patients harboring the p.Pro799Leu variant whose descriptions were limited to skeletal findings—the most common being progressive kyphoscoliosis, platyspondyly, shortening of long bones, and widened metaphysis [11,14,15,16,17] (Supplementary Table S1). A mosaic patient with the same variant showed an even milder phenotype (genu valgus, thickened articular cartilage of the knees, and irregular epiphysis) [18]. Importantly, these reports do not specify whether formal neurological evaluations were performed. The ClinVar database also includes a submission describing a patient with kyphoscoliosis, platyspondyly, motor axonal neuropathy, and muscle weakness, although no peer-reviewed publication for that case is available [13].

Combining our series with previously published cases, we identified 52 patients from 44 families with a documented clinical and molecular diagnosis of *TRPV4*-associated disorders; in four families, more than one affected individual was reported. Overall, 31 distinct pathogenic variants were detected, distributed across the *TRPV4* gene, with exon 14 harboring the largest fraction (6/31 variants, 19.4%). The two most frequent variants were p.Pro799Leu (9/44; 20.5%) and pArg594His (4/44; 9.1%). The recurrence of the p.Pro799Leu variant in multiple unrelated families [11,14,15] suggests it may represent a mutational hotspot.

The variants in our patients —p.Asn796del and p.Pro799Leu—are located within the carboxyl-terminal cytoplasmic domain of TRPV4 protein (Figure 2C), a region critical for interactions with regulatory proteins that modulate channel activity, oligomerization, and intracellular trafficking [6,14]. Functional assays demonstrate that p.Pro799Leu elevates basal intracellular calcium by constitutively activating the channel, thereby disrupting chondrocyte maturation and skeletal development [14,15].

Notably, clinical severity did not correlate predictably with specific variants: at least five variants have been reported in association with both isolated (skeletal-only or neurological-only) and complex (combined skeletal and neurological) phenotypes (p.Arg315Trp, p.Ser542Tyr, pArg594His, p.Leu618Pro, and p.Pro799Leu) (Supplementary Table S1), highlighting the need for careful clinical evaluation of both systems. Faye et al. [19] described a patient initially diagnosed with Spondylometaphyseal dysplasia-Kozlowski type based on radiographic findings, who later developed muscle weakness and gait abnormalities, leading to a revised diagnosis of CMT2C. The variant in that case, p.Arg315Trp (located in the ankyrin repeat domain), has been reported in isolated neuromuscular disease as well as in complex MD phenotypes, but not in purely skeletal forms [18,19,20]. Similarly, a family carrying p.Ser542Tyr was reported with six members diagnosed with CMT2C, four of whom exhibited a complex phenotype that included short stature likely due to underlying platyspondyly [20]. These observations support a dual pathogenic impact of some variants on peripheral nervous and skeletal development.

Several other variants have produced phenotypes similar to those of our patients 1 and 3. For example, Graversen et al. reported a patient with a pathogenic variant p.Gly280Ser who prenatally exhibited limb shortening and a bell-shaped thorax. Postnatally, she presented with a short neck, C2/C3 spinal stenosis, and tonic–clonic seizures. Neurological examination revealed reduced muscle strength and tone. At 21 months, she experienced cardiac arrest due to pneumonia complications and underwent surgical decompression for progressive myelopathy. Despite intervention, she developed a neurogenic bladder and severe obstipation [3].

Among the most severe presentations related to *TRPV4* variants is fetal akinesia. Unger et al. [21] reported four patients from three families (including one twin pregnancy) with reduced fetal movements and prenatal skeletal anomalies. Postmortem examination revealed severe platyspondyly in all cases, along with metaphyseal widening and shortened long bones in three patients. The identified variants (p.Gly78Trp, p.Lys276Glu, and p.Thr740Ile) have all been linked to MD and neuromuscular disease [21].

Although phenotype-modifying factors (epigenetic mechanisms, environmental exposures, and additional genetic variants) are frequently proposed to shape the clinical spectrum of TRPV4-associated disorders, empirical support remains limited. The most relevant data come from Woolums et al., who showed in Drosophila and in cultured primary mouse neurons that TRPV4-linked neurotoxicity is mediated by a Ca^2+^/calmodulin-dependent protein kinase II (CaMKII) mechanism and impairs axonal mitochondrial transport [22]. These findings nominate genes encoding components of CAMKII signaling and mitochondrial-trafficking pathways as plausible genetic modifiers of TRPV4 phenotypes, but targeted studies in humans are required to confirm their role.

This is the first report of metatropic dysplasia in the Mexican population. Given the restricted availability of advanced genomic testing in many settings, thorough clinical characterization, combined with targeted molecular analysis, remains essential for accurate diagnosis. Integrating clinical assessment with molecular data not only improves diagnostic certainty and genotype–phenotype correlation, but also informs prognosis, guides management, and enables appropriate genetic counseling for affected families.

This study has certain limitations, including the small cohort size and the restriction of molecular analysis to TRPV4 exon 14. These factors limit the generalizability of our observations and preclude a deeper investigation of potential genetic or epigenetic modifiers that may contribute to the observed phenotypic variability.

## 4. Conclusions

Our findings, together with previous reports, expand the phenotypic spectrum of metatropic dysplasia and reinforce a model in which TRPV4-related disorders lie along a gradient of clinical severity, rather than different syndromes. This variability appears to be influenced by the specific variant involved and potential genetic modifiers. We highlight the importance of early diagnosis and thorough clinical and molecular assessments to elucidate variant-specific effects on TRPV4 function. Such an approach will guide tailored multidisciplinary management, with the ultimate goal of mitigating complications and enhancing the quality of life for affected individuals.

## Data Availability

Data are contained within the article.

## Supplementary Materials

The following supporting information can be downloaded at: https://www.mdpi.com/article/10.3390/ijms26199783/s1: References [23,24,25,26,27,28,29] are cited in supplemental materials.
